# Longitudinal Study on Correlations Between Body Image, Physical Activity, and the Subjective Well-Being Among Adolescents Aged 14–16

**DOI:** 10.1177/10598405231191281

**Published:** 2023-07-31

**Authors:** Noora Matilainen, Hugo Blomberg, Ann-Christin Sollerhed, Eva-Lena Einberg, Pernilla Garmy

**Affiliations:** 1 Department of Nursing and Health Sciences, Faculty of Health Sciences, 4342 Kristianstad University, Kristianstad, Sweden; 2 Department of Primary Teacher Education, Faculty of Education, 4342 Kristianstad University, Kristianstad, Sweden; 3 Department of Health Sciences, Faculty of Medicine, 5193 Lund University, Lund, Sweden

**Keywords:** body image, body functioning, body appearance, physical activity, subjective well-being, perceived well-being, adolescents

## Abstract

This study examined the relationship between physical activity, body image, and subjective well-being among Swedish adolescents over time. Surveying 2308 students, with 137 providing longitudinal data, we conducted a multivariate logistic regression analysis. No significant correlations were found between physical activity (*p* = .268), body functioning (*p* = .567), or body appearance (*p* = .075) at age 14 and subjective well-being at age 16. Among control variables, sex (*p* = .038) and subjective well-being at age 14 (*p* = .013) showed significant correlations, while economic status did not (*p* = .39). The correlation between a positive subjective well-being at age 14 and age 16 indicates the importance of impacting the sense of well-being early. Further longitudinal studies are needed to explore the potential long-term correlation between body image and adolescent subjective well-being.

## Introduction

The World Health Organization (WHO, 2020) and The Public Health Agency of Sweden (2018) have recognized the significance of physical activity and body image during adolescence for long-term health and well-being. The theory of self-efficacy (Bandura, 1997) encompasses a person's confidence in her/himself and could be used to understand the possible link between physical activity, body image, and well-being. Swedish school nurses are expected to possess knowledge of risk factors for poor health and engage in preventative work and health promotion based on scientific evidence (Swedish National Association for School Nurses and Swedish Association for Nurses, 2016). To prevent poor health and to promote health and well-being, it is, therefore, good to assess the levels of physical activity and body image among adolescents and investigate potential correlations with perceived well-being over time.

Adolescence, spanning from 10 to 19 years of age as defined by the WHO (2022), is characterized by significant physical, cognitive, and psychosocial development. Mental well-being lacks a consensus definition, but according to the Swedish Public Health Agency (2018), it refers to a state where individuals can fulfill their potential, manage normal stress, work productively, and contribute to society. Results from the 2017 Health Behaviour in School-aged Children (HBSC) survey revealed that 66% of Swedish 15-year-olds reported “good” or “very good” well-being, with boys reporting higher well-being than girls. Girls were more likely to rate their well-being as “bad or very bad” (Swedish Public Health Agency, 2018). Family financial status is an essential factor influencing adolescents’ perceived well-being, as demonstrated by a cross-sectional study of Swedish adolescents by Plenty and Mood (2016), which also identified social status among peers as an equally important factor. Regulations and goals for Swedish school health services emphasize promoting mental health and early detection of students who may not be faring well.

Extensive research has examined the correlation between physical activity and better health among adolescents, both in terms of physical aspects, such as cardiovascular and metabolic functioning, and mental well-being (Ames & Leadbeater, 2018; Mountjoy et al., 2011; WHO, 2020). A European cross-sectional study by Granger et al. (2017) involving a large sample of 15-year-olds (*n* = 13,783) supports this correlation. With only 13.3% of the cohort meeting the WHO (2020) targets for physical activity for 5–17 year-olds, these targets require more intense aerobic exercise containing some muscle straining three times per week and some form of general physical activity for an average of 60 min per day (WHO, 2020). Longitudinal cohort studies, such as the one conducted by Ames and Leadbeater (2018) in Canada with 662 participants, indicate that the level of physical activity in youth is associated with the level of depressive symptoms as a young adult. Additionally, Dyremyhr et al. (2014) conducted a cross-sectional study of 2,527 adolescents in a Norwegian town, with 67% aged between 15 and 17 years old, and found a positive correlation between physical activity and perceived health. However, their study also found that highly physically active adolescents, particularly girls involved in team sports, had a greater risk of negative body image. This paradox highlights the need to consider the effects of physical activity on body image, which correlates with future well-being and morbidity when designing health promotion interventions targeting adolescents.

During adolescence, individuals undergo significant physical changes and experience mental processes that make them more aware of their bodies and more inclined to compare themselves with others. This period is crucial for an individual's sense of body image, and older adolescents tend to have a more negative body image than when they were in early adolescence (Swedish Public Health Agency, 2018). Meland et al. (2007) confirmed that girls and older participants in their study reported greater dissatisfaction with their weight and appearance than boys and younger participants. Negative body image is significantly correlated with lower levels of perceived well-being, especially among girls.

Research indicates a correlation between physical activity and body image. A newly published review by Foley Davelaar (2021) reveals that body image is a more important factor than skill level in adolescents’ decisions to quit youth sports at around 13 years of age. Berg and Larsson (2020) examined Swedish results from HBSC 1994–2014 and showed that efforts to lose weight had increased during the period, and dissatisfaction with one's weight was connected to lower levels of perceived health and other negative aspects. Therefore, promoting healthy body image for girls and boys during their school years may support their well-being and overall health.

The WHO (2021) highlights the potential of school health services to positively impact adolescent well-being, including in areas such as physical activity and body image. Langford et al. (2014) aimed to assess the effectiveness of the WHO's Health Promoting Schools (HPS) framework in improving the health and well-being of students and their academic achievement. The review provided evidence for the effectiveness of some interventions based on the HPS framework for improving certain health outcomes but not others (Langford et al., 2014). In Sweden, school health services are staffed by a range of professionals, including school nurses, physicians, counselors, special education teachers, and psychologists, who focus on risk and protective factors (National Board of Health and Welfare & Swedish National Agency for Education, 2016). As part of their work, school nurses engage in regular health conversations with students, covering topics such as physical activity and body image.

Previous cross-sectional studies in Sweden have demonstrated correlations between physical activity and body image in early adolescence and perceived well-being (Sollerhed et al., 2021, 2022). Longitudinal studies conducted internationally suggest that body image in adolescence can predict perceived well-being later in life (Gestsdottir et al., 2017) and that physical activity levels are associated with later mental health (Ames & Leadbeater, 2018). However, there is a lack of longitudinal studies conducted in the Swedish context to determine if these correlations hold in that population.

The primary objective of this study was to investigate potential correlations between physical activity levels and body image in 14-year-old Swedish adolescents and their subjective well-being when they were 16 years old, with sex, perceived family financial situation, and perceived well-being at 14 years old serving as control variables. The secondary aim was to explore how these variables developed between the ages of 14 and 16.

## Methods

The current study employed a non-experimental prospective design and utilized survey data to explore the potential correlation between body image, physical activity, and perceived well-being over time. Ethics approval for the project was granted by the Regional Review Board, and the study adhered to the human rights principles outlined in the Declaration of Helsinki. Before participating in the study, participants and their parents or legal guardians received written and oral information about the study, and informed consent was obtained. Participation was voluntary, and participants had the right to withdraw at any time.

Data for this study were collected from two surveys administered to schoolchildren in a southern municipality in Sweden, either during their individual visits to the school nurse or through an online questionnaire. The first survey was conducted between 2015 and 2017 and included students in grade 8. The second survey, which was conducted between 2017 and 2019, included first-year students from four participating upper secondary schools within the same municipality. Only data from this specific municipality was used in the present study.

The questionnaire items used in this study were identical in both surveys and focused on body image, physical activity, and subjective well-being, while background variables included sex and perceived family financial situation. The body image items measured satisfaction with the appearance and functioning of the body and were referred to as “body appearance” and “body functioning.” The questionnaire has been used earlier and has been found to be reliable for this age group (Sollerhed, 2006).

### Context

This study took part in a southern Swedish municipality with a population of approximately 130,000 as of 2021. The municipality contains a university with a relatively younger population than the Swedish average.

### Sample

**Figure 1.**
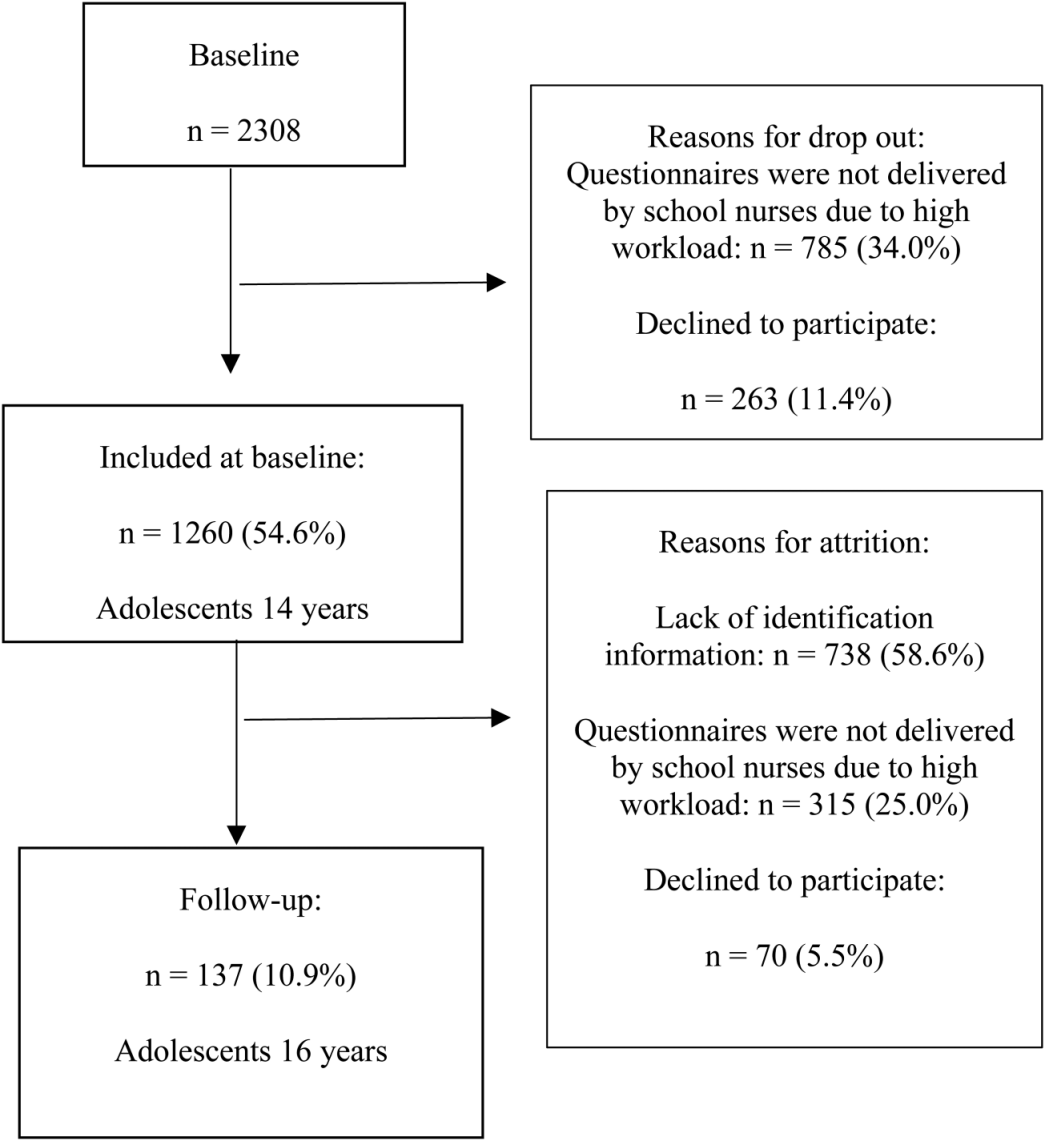
Selection process at baseline and follow-up.

The study targeted all 2,308 grade 8 students in the included municipality, and again 2 years later. Respondents had the option to submit their responses anonymously or with their names attached. For this study, a subset of participants (*n* = 137) who responded with their names on both occasions was included, that is, to track longitudinal data. Figure 1 provides a graphical representation of the included surveys and attrition.

### Statistical Analysis

The data used in this study was obtained from the two surveys and analyzed using IBM SPSS Version 25. Descriptive statistics were conducted to provide an overview of the sample group, including sex, perceived family financial situation, physical activity, body appearance, body functioning, and well-being. Sex and financial situation were included as variables due to their potential impact on adolescents’ perceived well-being, as indicated by prior research (Landstedt & Gådin, 2012; Plenty & Mood, 2016; Wiklund et al., 2012; The Public Health Agency of Sweden, 2015, 2018). Perceived well-being at age 14 was included as a control variable for the outcome at age 16.

To identify any correlations between variables, bivariate and multiple regression analyses were conducted. The dependent variable was perceived well-being at age 16, while the independent variables at age 14 included physical activity, body image, sex, perceived family financial situation, and perceived well-being at age 14. Additionally, bivariate regression analyses were performed for each variable separately to detect any changes over the 2 years from age 14 to 16. Chi-square tests and multiple logistic regression analyses were used for the bivariate and multivariate analyses, respectively. To enable multiple logistic regression analysis, perceived well-being at age 16 was dichotomized into two groups: one group that replied, “very good” or “quite good” and another group that replied “neither good nor bad,” “quite bad,” or “very bad.” This dichotomization was also applied in the bivariate tests. All independent variables were included in the analysis without being dichotomized. Results with *p* < .05 were considered significant.

## Results

Table 1 shows the sex distribution in the sample, which was slightly skewed, with 56.2% being female. Additionally, most participants perceived their family's financial situation as either very good or good.

**Table 1.** Background Characteristics.

Background Variables	*n* (%)
**Sex**	
Boys	60 (43.8)
Girls	77 (56.2)
Other	-
**Perceived family financial situation**	
Very good	67 (48.9)
Quite good	44 (32.1)
Average	25 (18.2)
Not so good	-
Not good at all	-
Don’t know	1 (0.7)

Table 2 presents the descriptive statistics for the distribution of responses. Overall, the students reported positive evaluations of their perceived well-being, body image (including appearance and functioning), and physical activity in both surveys. However, a significant decline was observed in all variables from the age of 14 to the age of 16 (*p* < .001 for all).

**Table 2.** Distribution of Responses at 14 and 16 Years of Age.

Variables	Answer at the Age of 14 *n* (%)	Answer at the Age of 16 *n* (%)
**Physical activity (PA)**		
Regularly four times a week or more	48 (35)	39 (28.5)
Regularly three times a week	29 (21.2)	32 (23.4)
Regularly two times a week	26 (19.0)	26 (19.0)
Regularly once a week	14 (10.2)	15 (10.9)
About once per month	11 (8.0)	16 (11.7)
About once per year	4 (2.9)	6 (4.4)
Never	5 (3.6)	3 (2.2)
**Perceived body appearance**		
Yes, completely satisfied	33 (24.1)	16 (11.7)
Yes, quite satisfied	81 (59.1)	82 (59.9)
No, quite dissatisfied	18 (13.1)	28 (20.4)
No, not at all satisfied	5 (3.6)	11 (8.0)
**Perceived body functioning**		
Yes, completely satisfied	59 (43.1)	40 (29.2)
Yes, quite satisfied	70 (51.1)	75 (54.7)
No, quite dissatisfied	8 (5.8)	16 (11.7)
No, not at all satisfied	-	4 (8.0)
**Perceived well-being**		
Very good	60 (43.8)	29 (21.2)
Quite good	63 (46.0)	72 (52.6)
Neither good nor bad	12 (8.8)	27 (19.7)
Quite bad	1 (0.7)	7 (5.1)
Very bad	-	2 (1.5)

The results of the bivariate analysis of the independent variables (at age 14) and background variables in relation to the outcome (perceived well-being at age 16) are shown in Table 3. All of these analyses produced significant results, except for physical activity at age 14, which did not show a significant relationship with perceived well-being at age 16 (*p* = .269).

**Table 3.** Bivariate Analysis of Perceived well-Being at the Age of 16 and Factors at the Age of 14.

Variables	Very Good or Quite Good Perceived Well-Being at the Age of 16 (*n* = 101), *n* (%)	Neither Good nor Bad, Quite Bad or Very Bad Perceived Well-Being at the Age of 16 (*n* = 36), *n* (%)	*p*-Value
**Sex**			.002*
Boys	52 (51.5)	8 (22.2)	
Girls	49 (48.5)	28 (77.8)	
**Perceived family financial situation**			.031*
Very good	55 (54.5)	12 (33.3)	
Quite good	32 (31.7)	12 (33.3)	
Average	13 (12.9)	12 (33.3)	
Not so good	-	-	
Not good at all	-	-	
Do not know	1 (1)		
**Physical activity at the age of 14**			.269
Regularly four times a week or more	38 (37.6)	10 (27.8)	
Regularly three times a week	18 (17.8)	11 (30.6)	
Regularly two times a week	21 (20.8)	5 (13.9)	
Regularly once a week	11 (10.9)	3 (8.3)	
Sometime per month	6 (5.9)	5 (13.9)	
Sometime per year	4 (4.0)	-	
Never	3 (3.0)	2 (5.6)	
**Perceived body appearance at the age of 14**			<.001*
Yes, completely satisfied	27 (26.7)	6 (16.7)	
Yes, quite satisfied	66 (65.3)	15 (41.7)	
No, quite dissatisfied	8 (7.9)	10 (27.8)	
No, not at all satisfied	-	5 (13.9)	
**Perceived body functioning at the age of 14**			.003*
Yes, completely satisfied	51 (50.5)	8 (22.2)	
Yes, quite satisfied	47 (46.5)	23 (63.9)	
No, quite dissatisfied	3 (3)	5 (13.9)	
No, not at all satisfied	-	-	

*p < .05.

The results of the multivariate analysis are presented in Table 4. The analysis revealed significant associations between sex (*p* = .038) and perceived well-being at age 14 (*p* = .013) with the dependent variable, perceived well-being at age 16. None of the independent variables in the primary aim of this study showed a significant correlation with the outcome. However, it is worth noting that body appearance exhibited a trend toward significance (*p* = .075).

**Table 4.** Multiple Regression Analysis of the Dependent Variable Perceived Well-Being at 16 Years Old and the Independent Variables, Sex, Perceived Family Financial Situation, Perceived Body Appearance and Body Functioning and Perceived Well-Being at 14 Years Old.

Independent Variables	OR	95% CI	*p*
Sex	2.767	1.058–7.237	.038*
Perceived family financial situation	1.275	0.733–2.219	.390
Physical activity	1.173	0.885–1.554	.268
Perceived body appearance	1.954	0.934–4.089	.075
Perceived body functioning	1.311	0.519–3.311	.567
Perceived well-being at the age of 14	2.666	1.232–5.768	.013*

OR = odds ratio; CI = confidence interval. Hosmer-Lemeshow goodness-of-fit test, *p* = .223, Nagelkerke pseudo *R*² = 0.306.

*p < .05.

## Discussion

The main finding was that after controlling for other variables such as sex and previous perceived well-being, there was no significant correlation between physical activity and body image at age 14 and perceived well-being at age 16. However, significant correlations between sex and previous perceived well-being with perceived well-being at age 16 were found. These results support that levels of physical activity, body image, and perceived well-being tend to be lower during later teenage years (age 16) compared to earlier teenage years (age 14).

The study found that physical activity at age 14 did not show a significant correlation with perceived well-being at age 16 in both bivariate and multivariate analyses. This finding contradicts previous cross-sectional research demonstrating a clear link between physical activity and well-being (Sollerhed et al., 2021, 2022). However, differences in study design, such as our longitudinal approach compared to previous cross-sectional studies and sample size, may explain this discrepancy. Bell et al. (2019) also found results similar to the present study, which showed no correlation between levels of physical activity at age 12–13 and mental well-being at age 15–16, although there was a correlation with “emotional problems.”

The present study confirms previous findings that physical activity levels tend to decrease as adolescents grow older and their participation in organized sports declines. This decline has been linked to increased levels of depressive symptoms, as demonstrated by Ames and Leadbeater (2018). Although it is not possible to draw any definite conclusions to explain the discrepancy in results between the present study and previous research, the findings suggest that study design, such as the longitudinal approach and sample size, may play a role.

In the bivariate analysis, body appearance at 14 clearly correlated with perceived well-being at 16. However, in the multivariate analysis, no significant correlation was observed. Despite this, the results were very close to being significant, indicating that further studies with larger sample size are needed before dismissing the hypothesis. Previous research suggests that a negative body image can lead to lower well-being in the long term. For example, the Swedish HBSC study from 2017/2018 found a correlation between negative body image and lower well-being (The Public Health Agency of Sweden, 2018). Gestsdottir et al. (2017) also found that negative body image can have a particularly detrimental effect on girls’ mental well-being later in life. Additionally, Lunde's (2009) doctoral thesis, which used a qualitative design, showed that negative body image could affect all aspects of life for some adolescents and severely impact their schooling and well-being.

A statistically significant correlation between sex and perceived well-being at the age of 16 was discovered in the present study. The results indicate that 77.8% (*n* = 28) of respondents who reported “neither good nor bad” or “bad” well-being were girls, which is consistent with previous research that suggests that teenage girls have lower well-being than boys of the same age. The Swedish section of the HBSC survey conducted in 2017 revealed that 15-year-old girls had lower levels of satisfaction with life, well-being, self-esteem, and self-efficacy than their male counterparts. A similar trend was observed among 13-year-olds but to a lesser extent. No clear sex difference was found among children aged 11. The HBSC survey also showed that girls reported more mental health and psychosomatic problems and were more likely to take medication for them. In a survey of Swedish adolescents aged 16–18, Wiklund et al. (2012) found that low perceived well-being and mental ill health were prevalent, especially among girls. Girls were more likely than boys to experience headaches, tiredness, trouble sleeping, muscular pains, depressive symptoms, and anxiety. Furthermore, 63.6% of girls reported stress and high demands from school, compared to 38.5% of boys. Landstedt and Gillander Gådin's study also confirmed that girls experienced more negative stress from stressors such as appearance and achievement.

Both bivariate and multivariate analyses revealed that the level of perceived well-being at the age of 14 was significantly correlated with perceived well-being at the age of 16. Despite a slight decline in overall perceived well-being at the group level, the current study found that higher levels of perceived well-being at age 14 significantly increased the likelihood of a similar outcome at age 16. This finding is consistent with Gustafsson et al.'s (2010) systematic review, which suggests that an individual's level of mental health tends to remain relatively stable throughout their school years, with some studies in the systematic review indicating that transitions such as entering upper secondary school can improve mental health. Additionally, several cross-sectional studies in the systematic review by Gustafsson et al.'s (2010) have shown lower levels of body image, physical activity, and perceived well-being in among older teens than younger teens. The present longitudinal study further confirms these findings by following the same individuals over time.

### Implications for School Nursing and Future Research

School nurses should prioritize promoting physical activity among adolescents due to the established link between physical activity and mental health in earlier cross-sectional studies in the same setting as the current study (Sollerhed et al., 2021, 2022). However, the findings in the current longitudinal study suggest that the positive effects of physical activity may not persist over prolonged periods. Therefore, school nurses must motivate adolescents to continue their physical activity to sustain its benefits. The results also emphasize the significance of early interventions by school nurses in promoting subjective well-being, as the strongest correlation was found between well-being in lower and upper secondary schools. School nurses can play a crucial role in promoting better well-being in adolescence, resulting in better health outcomes later in life.

Furthermore, the study emphasizes the need for further research on the correlation between body image and subjective well-being in adolescence. Future studies with larger sample sizes and longitudinal designs could provide more conclusive evidence of this correlation.

Finally, the study confirms the sex disparity in subjective well-being among adolescents, with girls reporting poorer well-being than boys. School nurses should consider this sex difference when providing care and support for adolescents. Further research is needed to better understand the underlying causes of this disparity and how school nurses can intervene to improve girls’ well-being.

### Limitations

The study has limitations in terms of its skewed perception of family financial status, limited sample size and attrition. These limitations should be considered when interpreting the study's results.
